# Central precocious puberty in Prader-Willi syndrome: a narrative review

**DOI:** 10.3389/fendo.2023.1150323

**Published:** 2023-05-08

**Authors:** Delia-Maria Nicoară, Alexandra-Cristina Scutca, Niculina Mang, Iulius Juganaru, Andrei-Ioan Munteanu, Luiza Vitan, Otilia Mărginean

**Affiliations:** ^1^ Department of Pediatrics, University of Medicine and Pharmacy “Victor Babes”, Timisoara, Romania; ^2^ Department of Pediatrics I, Children’s Emergency Hospital “Louis Turcanu”, Timisoara, Romania; ^3^ Research Center in Pediatrics - Disturbances of Growth and Development in Children – BELIVE, University of Medicine and Pharmacy “Victor Babes”, Timisoara, Romania; ^4^ Department of Endocrinology, Railway Hospital 2 Bucharest, Timisoara, Romania

**Keywords:** Prader-Willi syndrome, precocious puberty, endocrine, genetic, metabolic

## Abstract

Prader-Willi syndrome (PWS, OMIM176270) is a rare genetic disorder with recognizable dysmorphic features and multisystemic consequences such as endocrine, neurocognitive and metabolic ones. Although most patients with Prader-Willi syndrome exhibit hypogonadotropic hypogonadism, there is variability regarding sexual maturation, with precocious puberty occurring in rare cases. Our aim is to elaborate a thorough review of Prader-Willi patients with central precocious puberty, in order to raise awareness of such cases and to enhance our knowledge regarding the diagnosis and prompt treatment of this particular PWS patients.

## Introduction

Prader-Willi syndrome (PWS, OMIM176270) is a rare genetic disorder with recognizable dysmorphic features and multisystemic consequences such as endocrine, neurocognitive and metabolic ones (1, 2).

Although described in 1956 for the first time by Swiss endocrinologists Prader, Labhart and Willi (1), based on clinical aspects, genetic confirmation was possible only since 1980, when high resolution chromosome analysis led to the discovery of the chromosomal deletion of 15q11-g13

(3–5). Most PWS patients display a paternal microdeletion of the long arm of chromosome 15 (70%), whereas 20-35% have maternal uniparental disomy 15 (mUPD), and even fewer patients have an imprinting center defect (ICD) or an unbalanced translocation (0,1%) (6).

With an estimated prevalence of 1 in 10.000-30.000 live births, Prader-Willi syndrome represents the most common genetic cause of obesity (7–9) and the first recognized disorder of human genetic imprinting (10). PWS displays great clinical variability throughout life (1), ranging from hypotonia and failure to thrive during infancy, to morbid obesity, dysmorphic features, short stature and behavioral problems such as hyperphagia and aggressive behavior (11). Patients require multidisciplinary approach, including close endocrinologic follow-up throughout their lives (12) given the hypothalamic-pituitary dysfunction that characterizes these patients. This disfunction leads in turn to multiple endocrinopathies, the most common ones being growth hormone deficiency (GHD), hypogonadism and hypothyroidism (12, 13).

Genital anomalies are common in both female and male PWS patients and are represented by pubertal development disorders (14). Hypogonadism represents a major clinical diagnostic criteria of PWS, according to Holm and Cassidy (13). In Prader-Willi syndrome puberty is most often delayed and incomplete. While PWS male patients usually present cryptorchidism and remain in a Tanner genital stage II or III (14), most female PWS patients have amenorrhea or oligomenorrhea, with only a few undergoing spontaneous menarche (15). However, exceptional cases leading up to pregnancy have been described (6), isolated premature pubarche is reported in 14% of cases (1). Despite most PWS patients having hypogonadism, a few cases of central precocious puberty (CPP) have been reported (16).

Our aim is to elaborate a thorough review of Prader-Willi patients that display central precocious puberty, in order to raise awareness of such cases and to enhance our knowledge regarding the diagnosis and prompt treatment of this particular PWS patients.

## Materials and methods

### Search strategy

• The literature review was based on the available papers written in English published in the electronic databases PubMed and Embase database between 1979 and January 2023.
• The search criteria used in the Medical Subject Headings (MeSH) included te following terms: “Prader-Willi” [All Fields] AND (“precocious puberty)”[MeSH Terms]
• RCTs reported as literature reviews, case reports and conference abstracts with relevant outcome data were included in the review. Articles and abstracts regarding only premature adrenarche, or cases without genetic confirmation were excluded from the study.
• Duplicates were removed.

### Presentation of the results

The following data were collected from each article/abstract if available: age and gender, genetic background, Tanner Pubertal Stage, GnRH stimulation test, determination of basal and peak Luteinizing Hormone (LH) and Follicle Stimulating Hormone (FSH) serum levels, Testosterone, bone age, ultrasound of genitalia, stabilization or regression of Tanner Pubertal Stage under GnRH (gonadotropin releasing hormone) treatment according to dose and duration.

Patients were included if they fulfilled diagnostic criteria for CPP (i.e. age at pubertal onset, bone age, Tanner stage, significant value of LH, FSH and Testosterone GnRH stimulation test).

## Results

We sought to summarize the following outcome measures from the reviewed articles:

▪ suppression of physical signs of puberty: breast development in females, testicular volume in males, genital development – based on modified Tanner staging
▪ incremental growth rate: cm/year
▪ suppression of GnRHa – stimulated LH: peak LH value
▪ incremental change in bone age

We identified a total of 22 children with CPP and genetically confirmed Prader-Willi syndrome mostly in case reports and abstracts, summarizing 13 children from case reports and case studies [13-22] and 9 children from Embase indexed abstracts [23-27]. There were 13 (59%) male patients and 9 (41%) female cases, with mean age at CPP onset of 7,64 +/- 1,01 years (5 – 9 years). At diagnosis they were Tanner stage II-III, mean basal LH was 1,43 +/- 1,46 UI/L (0,3 – 4,6 UI/L), and mean stimulated peak LH was 13,67 +/- 6,45 UI/L (4,6 – 29,7 UI/L). Mean basal FSH was 1,43 +/- 1,46 UI/L (0,3 – 4,6 UI/L) stimulated peak FSH was 13,67 +/- 6,45 UI/L (4,6 – 29,7 UI/L).

Auxological parameters and response to GnRH of the PWS patients with precocious puberty are displayed below in **Tables 1**, **2**.

**Table 1:** Characteristics of PWS patients with central precocious puberty retrieved from case presentations/case studies.

Reference	Gender	Age at CPP onset (years)	Clinical signs	Growth velocity (cm/year)	Basal/stimulated peak LH	Basal/stimulated peak FSH	Oestradiol/Testosterone (before/during LNRH)	Bone age (years)	Cerebral MRI
Crinò et al. (13)*, 2008*	M	8,9	Tanner stage II	7	0,4/15,3	5,8/10,9	6.98mmol/L/0,61-1,6 mmol/L	10,6	Gliotic ischemia - left subcortical parietooccipital area
Ludwig et al. (17), 2016	M	8,8	Tanner stage II	NM	0,5/15,8	5,6/NM	54.7 ng/dL/18,7 ng/dL	9	No abnormalities
Monai et al. (18), 2019	M	8,2	Tanner stage II Testicular volume - 3 mL	7,1	1,1/11,1	5,2/7	0.45 nmol/L/NM	9,8	NM
Monai et al. (18), 2019	M	7,2	Tanner stage III (PH) Testicular volume - 4 mL	NM	NM/8,5	NM/3	1.25 nmol/L/NM	NM	NM
Kobayashi et al. (16), 2022	M	7,2	Tanner stage III (PH) Testicular volume - 6 mL	NM	0,5/29,7	5/22,6	0,41 ng/mL/NM	12,5	No abnormalities
Lee et al. (15), 2013	F	8,2	Genitalia stage (Tanner) III Pubic hair (Tanner) I	7	1/10,3	1,7/9,2	15 pg/mL (↑)/N	10,5	NM
Linnemann et al. (19), 1999	M	6,6	Tanner stage II (PH) Testicular volume - 5 mL	NM	0,5/5	1,6/3,6	2.4 nmol/L(↑) /2.1 nmol/L (↑)	9,1	Flat, small pituitary gland
Pusz et al. (20), 2008	F	5	Tanner stage III	NM	4,6	14,8	23 pg/mL (N)	13,6 (9,4)	No abnormalities
Crino et al. (21), 2003	F	7,2	NM	NM	NM	NM	NM	NM	NM
Crino et al. (21), 2003	F	7,4	NM	NM	NM	NM	NM	NM	NM
Crino et al. (21), 2003	M	8	NM	NM	NM	NM	NM	NM	NM
Eldar-Geva et al. (14), 2010	F	< 8	NM	NM	3,3/13,5	10,7/14,7	NM	NM	NM
Hirsch et al. (22), 2009	M	7	NM	NM	2,1/16,3	3,5/5,3	NM	11 (8,5)	5-mm diameter pineal cyst
Reference	Gender	Age at start of GnRH analogs (years)	Type/dose of GnRH product			Age at discontinuation of GnRH analogs (years)	Stabilization/regression of Tanner Pubertal Stage with GnRH analogs		
Crinò et al. (13)*, 2008*	M	8,9	Leuprorelin 3,75mg IM/28days			11,3	Good clinical and hormonal response already evident after 4 months		
Ludwig et al. (17), 2016	M	8,8	Leuprorelin 3,75mg IM/28days, than 11,25mg/3m			13	Satisfactory response, no progression of bone age and pubertal Tanner stage III, spontaneous resolution of testicular size discrepancy		
Monai et al. (18), 2019	M	8,2	Leuprorelin 11.25 mg S.Q./3m			12,3	still prepubertal at 12,8 years		
Monai et al. (18), 2019	M	7,4	Leuprorelin 3,75mg/28days			11,5	normal progression in growth and puberty, at 14 years he was fully virilized with testicular volume of 10 mL		
Kobayashi et al. (16), 2022	M	7,5	Leuprorelin acetate 0.94 mg S.Q./28days			11,3	Right and left testicular volume - 10 mL and 15 mL, respectively, Tanner stage 5 pubic hair		
Lee et al. (15), 2013	F	8,2	Leuprorelin 3.75 mg S.Q./28 days			NM	Regression of breast development noted after 3 months, stabilization of bone age		
Linnemann et al. (19), 1999	M	NO	NO			NO	Testicular volume and pubic hair developed slowly		
Pusz et al. (20), 2008	F	8,8	LHRH analog Depot 11.25 mg/28 days for months While on monthly LHRH analog she had persistent unexplained fevers and was changed to daily LHRH analog injection of 0.3 mg.			NM	Menstruation was suppressed, breast development regressed to Tanner II		
Crino et al. (21), 2003	F	7,2	NM			NM	Tanner stage 4 breast and pubic hair, menstruation at 10.2 years		
Crino et al. (21), 2003	F	7,4	NM			NM	Tanner stage 3 breast, Tanner stage 2 pubic hair		
Crino et al. (21), 2003	M	8	NM			NM	Tanner stage 2 genitalia, Tanner stage 3 pubic hair		
Eldar-Geva et al. (14), 2010	F	< 8	NM			NM	NM		
Hirsch et al. (22), 2009	M	7	NM			NM	NM		
Reference	Gender	Age at diagnostic (years)	Comorbidities			GH treatment	Years of follow- up			
Crinò et al. *(* 13)*, 2008*	M	5,2	Mild perinatal hypoxic damage Seizures at 4 years			1O years -> 15,3 years	8			
Ludwig et al. (17), 2016	M	6	NM			7 years -> NM	7			
Monai et al. (18), 2019	M	< 1	NM			8 months -> NM	4,5			
Monai et al. *(* 18), 2019	M	< 1	NM			2 years -> NM	NM			
Kobayashi et al. (16), 2022	M	10	NM			yes	8			
Lee et al. *(* 15), 2013	F	< 1	NM			3 years -> last follow-up	1,3			
Linnemann et al. (19), 1999	M	5	Forceps extraction because of intrauterine asphyxia			NM	NM			
Pusz et al. (20), 2008	F	8	Hypothyroidism, osteopenia, Raynaud’s phenomenon			yes	9,4			
Crino et al. (21), 2003	F	NM	NM			yes	5, 6			
Crino et al. (21), 2003	F	NM	NM			yes	3			
Crino et al. (21), 2003	M	NM	NM			yes	2,6			
Eldar-Geva et al. (14), 2010	F	NM	NM			NM	NM			
Hirsch et al. (22), 2009	M	NM	NM			NM	NM			

NM, not mentioned.

**Table 2:** Characteristics of PWS patients with central precocious puberty retrieved from case conference abstracts.

Reference	Gender	Age at diagnostic	Comorbidities				GH treatment		Years of follow- up	
Pellegrin et al *(* 23), 2016	M	NM	Hypothyroidism since age 2				1,5 years -> NM		NM	
Papagianni et al *(* 24), 2016	F	2 months	NM				NM		NM	
Karachaliou et al *(* 25), 2013	M	NM	hypobetalipoproteinemia				NM		NM	
Cheon et al. *(* 26), 2017	2M/3F	NM	NM				NM		NM	
Lu et al. *(* 27), 2019	M	NM	Epilepsy since age 4				yes		NM	
Reference	Gende r	Age at start of GnRH analogs (years)	Type/dose of GnRh product				Age at discontin uation of GnRH analogs (years)		Stabilization/regression of Tanner Pubertal Stage with GnRH analogs	
Pellegrin et al *(* 23), 2016	M	8,5	LHRH analogue				NM		good clinical and hormonal response	
Papagianni et al *(* 24), 2016	F	7,6	LHRH analogue				NM		NM	
Karachaliou et al *(* 25), 2013	M	9	NM				NM		suppressed pubertal development	
Cheon et al. *(* 26), 2017	2M/3F	NM	NM				NM		good clinical and hormonal response	
Lu et al. *(* 27), 2019	M	NM	NM				NM		NM	
Reference	Gender	Age at CPP onset (years)	Clinical signs	Growt h velocity (cm/ye ar)	Basal/stimulate d peak LH	Basal/stimulate d peak FSH	Oestradiol/Testosterone (before/duri ng LNRH)	Bone age (years)	Cerebral MRI
Pellegrin et al *(* 23), 2016	M	8,5	Tanner stage II for pubic hair and genitalia volume of testis 5 ml	9.4	NM/11.6 mUI/ml	NM/10.8 mUI/ml	1.15 ng/ml	10	Mild hydrocephalus (neonatal intraventricular haemorrhage), normal pituitary gland
Papagianni et al. (24), 2016	F	7,6	Tanner stage: B2A1P2M0	NM	NM/17.4 mIU/ml	NM/13.4 mIU/ml	↑	NM	normal
Karachaliou et al. (25), 2013	M	9	BMISDS: 1.61, pubic hair (PH) 2, testes 5ml	7,5	NM/ ↑	NM/ ↑	NM	10	normal
Cheon et al. *(* 26), 2017	2M/3F	NM	Tanner stage II for breast/testicular development and a Tanner stage I for pubic hair development	8.7 ± 0.6	0.3 ± 0.3 IU/L /11.8 ± 8.0 IU/L	3.7 ± 2.9 IU/Ml /NM	NM	↑ by 2.6 ± 0.5 years	Normal pituitary MRI
Lu et al. *(* 27), 2019	M	~ 6	increased testicular volume and growth velocity	NM	NM/20.51 mIU/mL	NM	3.32 nmol/L	↑	NM

NM, not mentioned. ↑ elevated.

## Discussions

Pubertal timing is a complex process, with genetic, epigenetic, endocrine, metabolic and lifestyle factors playing a role in acquiring secondary sexual characteristics, gonadal maturation and progression of linear growth (28, 29). It normally coincides with the pulsatile release of GnRH from the hypothalamic neurons (30). Normal pulsatile GnRH secretion is initially observed during fetal and neonatal periods (31, 32), suppressed during childhood, only to resume at the age of puberty, under action of Kisspeptin (33).

The first symptoms of precocious puberty are regarded in girls as onset of breast development before the age of 8 years, and in boys as testicular enlargement ≥ 4 ml before the age of 9 years (29), in accordance with the mean age at CPP onset in the reviewed cases (7,64 +/- 1,01 years). The underlying mechanism can be central (GnRH- dependent) or peripheral (GnRH-independent). In Prader-Willi cases associated with precocious puberty only central precocious puberty has been described (13–27).

## Laboratory and imagistic evaluation

The most sensitive biomarker in CPP is LH, as it is untraceable before the first stages of puberty (34). Therefore determining plasma LH after exogenous GnRH or LHRH stimulation represents the reference test for diagnosing central precocious puberty (35–38). Yet peak cut-off values beyond which puberty is activated remain controversial (35, 36, 38). Values between 5 and 10 IU/L are considered acceptable cut-off points, using Chemiluminescent immunoassay (36, 38). Further assessment includes assessment of bone age (usually advanced as compared to chronological age), pelvic or testicular ultrasonography (to aid in identifying signs of peripheral precocious puberty) and brain magnetic resonance imaging in order to evaluate the hypothalamus and pituitary glands, and to exclude other brain anomalies (39). 9 cases describe imagistic evaluation through cerebral MRI, out of which 3 had abnormalities: one male patient with ischemic cerebral lesions, one with pituitary hypoplasia and one with a small pineal cyst. It can be argued that such cerebral lesions account in part for the precocious puberal onset, but additional causes related to this patients remain obscure.

## Evolution and therapeutic approach

Premature activation of the hypothalamic–pituitary–gonadal (HPG) axis can have several repercussions in children and adolescents, from height deficit caused by premature fusion of epiphyses through accelerated bone maturation, to premature sexual maturation that cause body changes (34, 40). Accumulating evidence points out to the absolute or relative growth hormone deficiency in most cases of PWS patients (41–48), according to standard testing protocols (49). As such, adult height prognosis is additionally compromised in children with Prader-Willi syndrome that exhibit central precocious puberty. This reinforces the interest of detecting any central precocious puberty in Prader-Willi patients. Central precocious puberty also occurs in other diseases that associate hypopituitarism, among which lesions of the central nervous system, such as intracranial malignancies and cranial radiotherapy (50–53), severe head injury (53), arachnoid cyst and septo-optic dysplasia (54–58). Other possible situations involve genetic causes such as, combined pituitary hormone deficiency due to POU1F1 gene mutation (59, 60), Kabuki syndrome (61, 62), Williams–Beuren syndrome (63, 64), Mayer-Rokitansky-Kuster-Hauser syndrome (65) and developmental defect of the hypothalamic–pituitary area (66). The possible common etiological mechanisms in both situations should also be the subject of further studies, as the underlying mechanism of remains unclear (59).

Also, there is increased risk for metabolic comorbidities such as obesity, type 2 diabetes and for cardiovascular events (34, 67, 68). As such, there is increased need for prompt treatment. GnRH analogues are the cornerstone of treatment, and they are administered either intramuscular (1 administration every 28 days) or by subcutaneous implant (69). They have been used effectively in cases of CPP, reducing plasma gonadotrophins, gonadal steroids and peptides (34, 70). Adequate suppression of gonadotrophins can be seen after 3 – 4 months of treatment (71).

Future therapeutic options that are being evaluated as an alternative option include newer kisspeptin and neurokinin B antagonists (69, 72, 73). Clinical trials investigating the short term efficacy of GnRH analogs confirm the fact that this treatment is well tolerated and safe (71, 74–76). Also, case reports regarding PWS patients with central precocious puberty highlight the possible benefit of combined therapy (gonadotropin-releasing hormone agonist and recombinant human growth hormone) on final height, while restoring appropriate pubertal progression (16, 17).

## Study limitations

Due to the exceedingly rare cases of PWS patients with precocious puberty, reviewed cases stemmed from case reports and Embase registered conference abstracts. Yet, it is our belief that such cases promote a better understanding of the variety of sexual maturation disorders in PWS patients.

## Conclusions

Although most patients with Prader-Willi syndrome exhibit hypogonadotropic hypogonadism, there is variability regarding sexual maturation, with precocious puberty occurring in rare cases. Recent years have brought major improvement in scientific knowledge regarding PWS, but there is still a need for further studies to assess the pathophysiology implicated in timing and progression of pubertal onset (15), and to implement clinical guidelines (12). GnRH agonist therapy seems to be efficient and safe in such cases, although long term follow-up is of need to better address the issue (71, 74–76).

## Author contributions

DMN, ACS identified potential papers, D-MN and ACS wrote original draft, D-MN, A-CS, NM, IJ, A-IM and LV analyzed selected papers, and took the lead in writing the manuscript. OM supervised the project and recommended changes. All authors contributed to the article and approved the submitted version.
